# Editorial: Regulatory non-coding RNA networks in bone health and disease

**DOI:** 10.3389/fendo.2026.1939709

**Published:** 2026-07-27

**Authors:** Michela Rossi, Marta Gomarasca, Ettore Luzi

**Affiliations:** 1 Bone Physiopathology Research Unit, Translational Pediatrics and Clinical Genetics Research Division, Bambino Gesù Children’s Hospital, IRCCS, Rome, Italy; 2 Laboratory of Experimental Biochemistry and Advanced Diagnostics, IRCCS Ospedale Galeazzi-Sant’Ambrogio, Milano, Italy; 3 Virtual Laboratory of ncRNAs Complex Diseases University of Firenze (UNIFI), Firenze, Italy

**Keywords:** bone diseases, bone remodeling, miRNA, non-coding RNAs, osteoporosis

## Introduction

In recent years, non-coding RNA (ncRNA) have emerged as fundamental regulators of skeletal biology, profoundly transforming our understanding of bone development, remodeling, aging, and disease. Beyond their established role as post-transcriptional modulators of gene expression, microRNAs (miRNA), long non-coding RNA (lncRNA), and circular RNA (circRNA) are increasingly recognized as integral components of complex molecular networks governing osteoblast and osteoclast differentiation, osteocyte function, extracellular matrix remodeling, angiogenesis, inflammation, and intercellular communication (1). Advances in transcriptomics, epigenomics, and multi-omics technologies have greatly expanded our knowledge of ncRNA-mediated regulation and have revealed their involvement in nearly every aspect of skeletal physiology and pathology (2, 3).

Growing evidence indicates that ncRNA operate at the intersection of skeletal aging, osteoimmunology, mechanotransduction, and tissue regeneration. In particular, age-dependent accumulation of senescent cells, alterations in Wnt signaling, chronic low-grade inflammation, and impaired osteogenic potential have emerged as key mechanisms linking aging to osteoporosis and other musculoskeletal diseases (4–6). Simultaneously, extracellular vesicles and exosomes have been identified as important carriers of regulatory ncRNA, facilitating communication among osteoblasts, osteoclasts, stromal cells, endothelial cells, and immune cells (7). These discoveries have positioned ncRNA not only as mechanistic regulators but also as promising biomarkers and therapeutic targets for precision medicine approaches in skeletal disorders.

## MicroRNA regulation of bone remodeling and osteoporosis

Meng et al. provide a comprehensive and timely overview of the role of miRNA in osteoporosis. The authors describe how miRNA influence osteoblastogenesis, osteoclastogenesis, angiogenesis, and osteoimmunology through the modulation of major signaling pathways, including BMP/TGF-β, RANK-L/RANK/OPG, and Wnt/β-catenin. An important aspect of this review is the discussion of exosome-mediated miRNA transfer, highlighting the emerging concept that ncRNA function as mediators of communication within the bone microenvironment. Collectively, this work places miRNA at the center of bone remodeling networks and reinforces their potential as diagnostic and therapeutic tools.

## Non-coding RNA, skeletal aging and cellular senescence

The involvement of ncRNA in skeletal aging is explored experimentally by Singh et al., who identify miR-668-5p as a novel regulator of osteoblast function and age-related bone loss. By targeting the E3 ubiquitin ligase ZNRF3, miR-668-5p enhances Wnt/β-catenin signaling, promotes osteogenic differentiation, and attenuates cellular senescence and oxidative stress. The demonstration that modulation of a single miRNA can improve bone architecture and bone formation links ncRNA biology with the emerging field of gero-science and highlights the potential of RNA-based interventions for osteoporosis associated with aging or chemotherapy-induced damage.

## From molecular mechanisms to clinical biomarkers

Translational relevance is further emphasized by Li et al., who investigated circulating miRNA as biomarkers of diabetic osteoporosis. The identification of miR-188-3p, miR-335-5p, and miR-19a/b as serum signatures associated with bone mineral density extends the role of ncRNA beyond mechanistic studies into the clinical arena. Their findings support the notion that ncRNA profiling may contribute to early diagnosis, disease stratification, and patient-specific management in metabolic bone disease. Such approaches are particularly attractive because they complement conventional imaging and bone turnover markers with minimally invasive molecular tools.

## Non-coding RNAs in osteoarthritis: extending the ncRNA paradigm beyond bone

Jain et al. broaden the perspective of this Research Topic by reviewing ncRNA-mediated regulation in osteoarthritis. The authors summarize how miRNA, lncRNA, and circRNA influence cartilage degradation, synovial inflammation, osteoclastogenesis, and subchondral bone remodeling through pathways such as NF-kB, TGF-β/SMAD, PI3K/Akt, MAPK, and Wnt/β-catenin. Importantly, this review reinforces the modern view of osteoarthritis as a disease affecting the entire osteochondral unit and highlights ncRNA as potential biomarkers and therapeutic targets. The work also emphasizes emerging RNA-based strategies, including antisense oligonucleotides, miRNA mimics, exosome-based delivery platforms, and nanotechnologies.

## Conclusion

Collectively, the contributions gathered in this Research Topic illustrate the extraordinary versatility of ncRNA as regulators of musculoskeletal health and disease. From orchestrating bone remodeling and regulating skeletal aging to enabling biomarker discovery and therapeutic innovation, ncRNA are increasingly recognized as central determinants of tissue homeostasis. Although important challenges remain for clinical translation, particularly regarding delivery efficiency, tissue specificity, and long-term safety, continued progress in RNA biology and precision medicine is likely to accelerate their application in clinical practice. Together, these studies underscore the potential of ncRNA networks to reshape our understanding of osteoporosis, osteoarthritis, and other skeletal disorders while providing a foundation for future diagnostic and therapeutic advances.
